# Quality Control by Isoleucyl-tRNA Synthetase of *Bacillus subtilis* Is Required for Efficient Sporulation

**DOI:** 10.1038/srep41763

**Published:** 2017-01-31

**Authors:** Elizabeth Kermgard, Zhou Yang, Annika-Marisa Michel, Rachel Simari, Jacqueline Wong, Michael Ibba, Beth A. Lazazzera

**Affiliations:** 1 Department of Microbiology, Immunology and Molecular Genetics University of California, Los Angeles, California 90095, USA; 2 Technische Universität Braunschweig, Institut of Microbiology, Braunschweig, Germany; 3 Ohio State Biochemistry Program, Ohio State University, Columbus, Ohio 43210, USA; 4 Department of Microbiology, Ohio State University, Columbus, Ohio 43210, USA; 5 Center for RNA Biology, Ohio State University, Columbus, Ohio 43210, USA; 6 Molecular Biology Institute, University of California, Los Angeles, California 90095, USA

## Abstract

Isoleucyl-tRNA synthetase (IleRS) is an aminoacyl-tRNA synthetase whose essential function is to aminoacylate tRNA^Ile^ with isoleucine. Like some other aminoacyl-tRNA synthetases, IleRS can mischarge tRNA^Ile^ and correct this misacylation through a separate post-transfer editing function. To explore the biological significance of this editing function, we created a *ileS(T233P)* mutant of *Bacillus subtilis* that allows tRNA^Ile^ mischarging while retaining wild-type Ile-tRNA^Ile^ synthesis activity. As seen in other species defective for aminoacylation quality control, the growth rate of the *ileS(T233P)* strain was not significantly different from wild-type. When the *ileS(T233P)* strain was assessed for its ability to promote distinct phenotypes in response to starvation, the *ileS(T233P)* strain was observed to exhibit a significant defect in formation of environmentally resistant spores. The sporulation defect ranged from 3-fold to 30-fold and was due to a delay in activation of early sporulation genes. The loss of aminoacylation quality control in the *ileS(T233P)* strain resulted in the inability to compete with a wild-type strain under selective conditions that required sporulation. These data show that the quality control function of IleRS is required in *B. subtilis* for efficient sporulation and suggests that editing by aminoacyl-tRNA synthetases may be important for survival under starvation/nutrient limitation conditions.

An essential step for the accuracy of mRNA translation is the charging of tRNAs with their cognate amino acid. Aminoacyl-tRNA synthetases (aaRS) are the enzymes that catalyze this reaction, and are a family of ancient proteins that have been conserved throughout evolution, due to their essential cellular function^1^. AaRSs aminoacylate tRNAs through a two-step mechanism: 1) formation of an aminoacyl-adenylate (e.g. Ile-AMP by IleRS), and 2) aminoacylation of tRNA (e.g. Ile-tRNA^Ile^). Due to the need for accuracy in protein synthesis, some aaRSs contain a quality control (QC) function along with their aminoacylation function^2^. QC functions can hydrolyze either the aminoacyl-adenylate (pre-transfer editing) or the aminoacyl bond of charged tRNA (post-transfer editing). While aaRS editing functions are highly conserved in bacteria, archaea, and eukaryotes, QC is not essential for cellular growth under most laboratory conditions, and few studies have identified cellular functions that rely on aaRS editing^3^,^4^,^5^,^6^,^7^.

Isoleucine-tRNA synthetase (IleRS) possesses QC functions that discriminate between isoleucine, the non-cognate amino acid valine, and the non-proteinogenic amino acids, norvaline, a byproduct of branched-chain amino acid synthesis, and homocysteine (Hcy), a by-product from degradation of S-adenosylhomocysteine by LuxS in bacteria^8^. The aminoacylation site of IleRS is only able to discriminate Ile from Val with an accuracy of ~1/200^9^. IleRS improves this accuracy with two QC activities, pre- and post-transfer editing. Pre-transfer editing hydrolyzes Val-AMP in the synthetic site where aminoacylation also occurs^10^,^11^, and pre-transfer editing similarly hydrolyzes Hcy-AMP^12^. Post-transfer editing in contrast deacylates Val-tRNA^Ile^ in a domain, the connective peptide 1 (CP1) domain, which is distinct from the site at which aminoacylation occurs^13^,^14^,^15^,^16^. Several studies have identified substitutions in CP1 that disrupt post-transfer editing, but retain close to wild-type levels of aminoacylation activity^13^,^14^,^17^.

*In vivo* studies of IleRS mutants have begun to reveal potential cellular functions of QC by aaRSs. One primary function of quality control is to prevent misincorporation in proteins of the non-cognate and non-proteinogenic amino acids, such as valine and norvaline, respectively. Loss of IleRS QC in *E. coli* results in decreased growth when the cells are grown in high concentrations of valine or norvaline^18^. The *E. coli* IleRS QC mutant strain is also more sensitive to stressful conditions, including high temperatures and antibiotics, although the mechanism by which these mutant cells increase their sensitivity to stress is unknown^19^. Loss of quality control by IleRS may also be beneficial under certain conditions. In *E. coli* and *Acinetobacter baylyi*, loss of IleRS QC functions resulted in an improved growth rate when concentrations of isoleucine were limiting, but moderate concentrations of valine or norvaline were present^18^,^20^. *Streptococcus pneumoniae* IleRS naturally lacks a post-transfer editing function, and mischarged tRNA^Ile^ serves as a substrate for peptidoglycan biosynthesis^21^. Potentially beneficial is the higher level of mutations observed in colonies of *E. coli* IleRS QC-defective cells that are allowed to age; the full mechanism by which DNA mutations arise in response to loss of quality control is also unknown^22^. Overall, while a number of phenotypes have been described for QC mutants, the mechanism underlying the observed changes and their broader importance remain unknown.

To address the question of what conditions require quality control for fitness, we choose to address how loss of QC by IleRS affects the function of the model Gram-positive organism, *Bacillus subtilis*. Here, we show that QC by IleRS is required for *B. subtilis*, to form environmentally-resistant endospores. These endospores are formed in response to starvation through a developmental process. This process is initiated by a master regulator, Spo0A, and gene expression activated by this transcription factor is severely delayed in cells that lacking QC by IleRS. Furthermore, the QC-defective IleRS strain was outcompeted by wild-type strains under conditions involving periodic sporulation and outgrowth, supporting a general role for quality control by aaRS under starvation or nutrient-limited conditions.

## Results

### Cell expressing IleRS(T233P) are defective for quality control of mischarged tRNA^Ile^

IleRS was chosen for this study as it has a well characterized post-transfer editing activity, and substitutions that disrupt this editing without affecting aminoacylation have been made^10^,^11^,^23^. While several studies have looked at the role of QC by IleRS in *E. coli,* relatively few studies have examined the importance of QC in other organisms. As we had previously proposed that QC maybe more critical under conditions of stress that limit cellular growth^3^, we chose to analyze the role of QC by IleRS in *B.* subtilis, which undergoes several distinct phenotypic or developmental shifts in response to starvation or slow growth, including the formation of environmentally-resistant endospores^24^.

**Figure 1:**
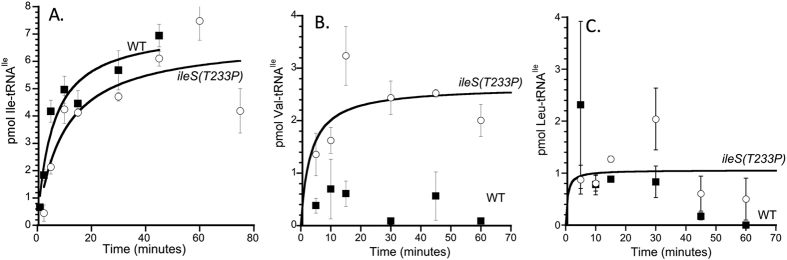
The *ileS(T233P)* strain mischarges tRNA^Ile^ with valine. Cell extracts of BAL4574 (WT; filled squares) and BAL4571(*ileS(T233P)*; open circles) were assayed for their ability to aminoacylate tRNA^Ile^ with ^14^C versions of isoleucine (**A**), valine (**B**), or leucine (**C**). Plotted is the average pmoles of the radiolabeled tRNA from three reactions versus the time after the addition of tRNA^Ile^ and the radiolabeled amino acid. Error bars are standard error of the mean. Lines are best fits of the data to a Michaelis-Menton equation, with the exception of WT in panels B and C, as the basal level of aminoacylation observed could not be fit.

To determine the function of QC by IleRS in *B. subtilis*, a single point mutation was introduced into the *ileS* gene on the chromosome of *B. subtilis* strain JH642^25^. The point mutation introduced causes a substitution of Thr at codon 233 to Pro. This T233P substitution was chosen because a similar substitution in IleRS of *Escherichia coli* was shown to disrupt QC by this enzyme, and while other substitutions in *E. coli* IleRS disrupt QC, the T233P substitution has virtually no effect on aminoacylation activity^14^,^23^. To test whether the cells with the *ileS(T233P)* allele lacked tRNA^Ile^ QC activity, cell lysates of the wild-type *ileS* and mutant *ileS(T233P*) were tested for their ability to charge tRNA^Ile^ with either the cognate Ile or the non-cognate amino acids, Val and Leu. Lysates from the *ileS(T233P)* strain showed Ile-tRNA^Ile^ production at a similar rate as lysates from wild-type cells (Fig. 1A), indicating that the *ileS(T233P)* mutation did not affect the overall level of Ile-tRNA^Ile^ formation in cells. Similarly, the *ileS(T233P)* lysate showed only low levels of Leu-tRNA^Ile^ production, similar to that seen with lysates from wild-type cells, and consistent with the Leu not being an aminoacylation substrate for either wild-type IleRS or IleRS(T233P) (Fig. 1C). Production of Val-tRNA^Ile^ however was significantly different between wild-type and *ileS(T233P)* cell lysates, with the *ileS(T233P)* lysate producing significantly more Val-tRNA^Ile^ than the wild-type lysate (Fig. 1B). Increased Val-tRNA^Ile^ synthesis for IleRS(T233P) may either indicate improved Val recognition and/or reduced hydrolysis of either misactivated Val-AMP or mischarged Val-tRNA^Ile^ compared with wild-type. Importantly, whatever the precise mechanism by which the T233P substitution increased Val-tRNA^Ile^ synthesis, this did not adversely affect the ability of cells to aminoacylate tRNA^Ile^ with its cognate amino acid and did disrupt QC by IleRS.

### Cells expressing IleRS(T233P) are defective in sporulation

Having created a mutant strain of *B. subtilis* that expresses an QC-defective version of IleRS, we sought to determine whether *ileS(T233P)* cells had any phenotypic differences from wild-type. Any phenotypes altered in these mutant cells would suggest pathways that are sensitive to QC by IleRS. The exponential growth rate (∆Ln OD_600_/∆time(min) ± standard deviation) in rich medium, Difco Sporulation Medium (DSM), of wild-type cells and cells expressing IleRS(T233P) were not significantly different (0.020 ± 0.002 and 0.021 ± 0.002, respectively). Similarly, in minimal medium, the exponential growth rate of these two strains was also not significantly different (0.011 ± 0.002 for wild-type cells and 0.010 ± 0.003 for the *ileS(T233P*) cells). These data are consistent with other observations of QC-defective aaRS bacterial strains are not affected for growth under standard laboratory conditions^3^.

As the editing function of IleRS is conserved through evolution^3^, and cells in natural environments are characterized by slow growth, the wild-type and *ileS(T233P)* mutant *B. subtilis* cells were assessed for their ability to form biofilms and spores, two major phenotypic states entered by this bacterium under slow-growth conditions^26^. While wild strains of *B. subtilis* form more robust biofilms, laboratory strain JH642 used in this study is able to form biofilms that adhere to the wells of a microtiter plate and to form flat, unstructured pellicle biofilms at air-liquid interfaces^27^,^28^,^29^,^30^. Both the *ileS(T233P)* strain and its isogenic wild-type strain formed morphologically indistinguishable pellicles that were observed as films of cells at the top of media in test tubes. In microtiter plates, there was at best a small difference in how the two strains adhered to the plates, and the *A*_570_ ± standard deviation was 1.4 ± 0.3 for the wild-type strain and 0.87 ± 0.02 for the *ileS(T233P)* strain. These data support a conclusion that the QC by IleRS is not essential for biofilm formation, although we cannot rule out a more prominent role for editing in wild strains of *B. subtilis*.

To assess the ability of the *ileS(T233P)* mutant cells to sporulate, these cells and the isogenic wild-type control cells were grown in DSM and incubated until sixteen hours after entry into stationary phase. At this time, the frequency of sporulation was determined as the number of heat-resistant colony-forming units (CFU)/ml relative to the total viable CFU/ml (Table 1). While the fold difference in the sporulation frequency of wild-type and the *ileS(T233P)* cells was variable, ranging from 1.5- to 15-fold, the *ileS(T233P)* cells showed a statistically significant (*p* = 0.015) defect in sporulation. The variability in the sporulation phenotype is not unexpected given the stochasticity in frequency of mischarging of a tRNA.

To determine whether this sporulation defect was specific to growth in DSM, cells were grown in minimal media containing 0.1% glucose. Cells in this media exhaust glucose and transition to stationary phase at an OD_600_ of ~1.0. After 16 hours in stationary phase, the frequency of sporulation was assayed (Table 1). Similar to DSM conditions, the *ileS(T233P)* mutant exhibited a statistically significant (*p* > 0.001) defect in sporulation, except that the *ileS(T233P)* cells showed a larger defect in sporulation, ranging from a 4- to 30-fold defect.

### The *ileS(T233P)* mutant is outcompeted by wild-type cells during cycles of sporulation

**Figure 2:**
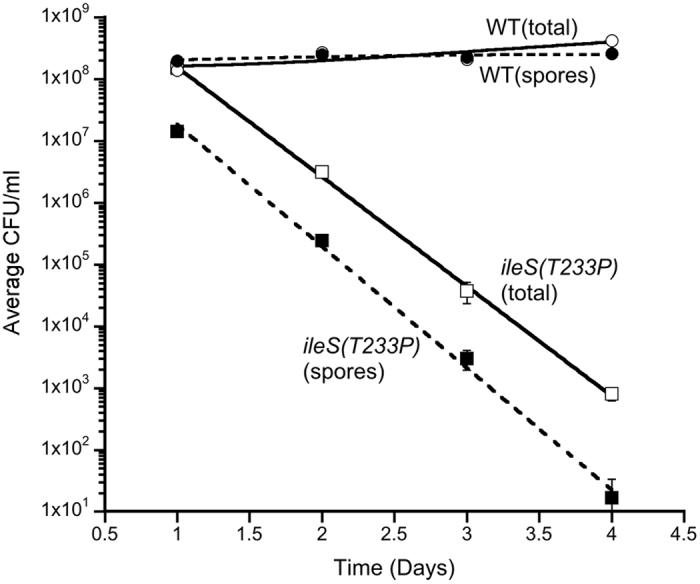
The *ileS(T233P)* strain is outcompeted by wild-type cells. Derivatives of BAL4574 (WT; circles) and BAL4571 (*ileS(T233P)*; squares), which carried either erythromycin-resistant or chloramphenicol-resistant genes, were mixed in equal numbers in DSM and incubated until 16 hours post the onset of stationary phase. At that time, the total CFU/ml (open symbols) was assessed for both cell types. The cultures were then heated to kill vegetative cells, and the CFU/ml of spores (filled symbols) was assessed for both cell types. A 1/10^th^ volume of the spores was then transferred to a new DSM culture, and the process was repeated for four days. Plotted is the average CFU/ml of four separate cultures versus the day of the competition. Error bars are standard error of the mean.

To assess the importance of the QC function of IleRS for *B. subtilis*, we asked how well the *ileS(T233P)* mutant was able to compete with the wild-type strain. We introduced into the wild-type and *ileS(T233P)* strains either a *thrC::erm* allele that encodes erythromycin resistance (Erm^R^) or a *thrC::cat* allele that encodes chloramphenicol resistance (Cm^R^). Cultures were inoculated with an equal mixture of both wild-type and *ileS(T233P)* cells of different antibiotic-resistance markers. In the repeats of the cultures, the antibiotic-resistance markers were swapped between the two strains to ensure that any differences observed were due to the presence of the *ileS(T233P)* mutation. The cultures were grown in DSM and passaged through a cycle of exponential growth, sporulation, and heat-kill of non-sporulated cells. A 1/10^th^ fraction of the spores that remained after the heat-kill step were used to inoculate the next culture that was similarly passaged through a cycle of exponential growth, sporulation, and heat-kill of non-sporulated cells. This was repeated for a total of four cycles. At the end of each cycle, the percent of the spores that were from wild-type and *ileS(T233P)* cells was measured through Cm^R^ and Erm^R^ CFU/ml, as appropriate. For each independent culture started, there was a similar number of cells of each strain at the end of the first passage, consistent with no significant differences in the growth rate of these two strains. Subsequently, the number of *ileS(T233P*) cells dropped relative to the number of wild-type cells by the second or third passage, with the level of *ileS(T233P*) mutant after the fourth passage being 10^5^-fold lower than wild-type (Fig. 2). Interestingly, under these competition experiments, the *ileS(T233P)* mutant consistently showed a greater that 10-fold defect in sporulation (Fig. 2), unlike what was observed for this strain when grown individually (Table 1). These data predict that the *ileS(T233P*) mutant would go to extinction after five cycles and strongly suggests that sporulation is one of the evolutionary pressures maintaining the QC function of IleRS in *B. subtilis*.

### The QC-defective IleRS mutant is defective in expressing genes activated by the master regulator of sporulation, Spo0A

A major question from this work is why sporulation is sensitive to mistakes made by IleRS. The process of sporulation can be divided into distinct genetic and morphological stages^31^. The sporulation defect of the *ileS(T233P)* could stem from mistranslation of Ile codons, which could result in a non-functional protein that is required for sporulation. If a particular protein is negatively affected in the *ileS(T233P)* strain, we would predict the *ileS(T233P)* cells to be specifically delayed at one stage of sporulation, resulting in a strong reduction in expression of those stage-specific genes. However, mistranslation is predicted to happen randomly at Ile codons, with each cell having a different mixture of mistranslated proteins, and as a result each cell in a population could be delayed at a different stage in sporulation, and we would not observe a strong effect on any particular stage-specific gene expression at the population level.

**Figure 3:**
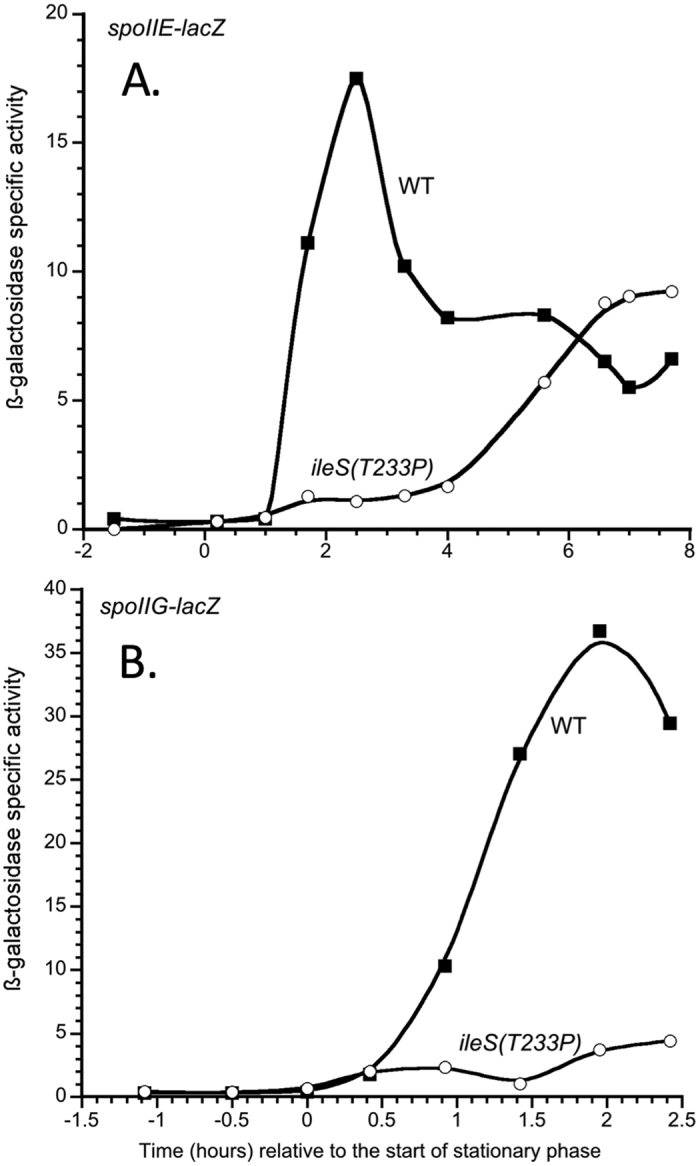
Expression of a Spo0A-activated gene is delayed in the *ileS(T233P)* mutant. Strains carrying a *spoIIE-lacZ* (**A**) or *spoIIG-lacZ* (**B**) fusion were grown in DSM. Samples were removed periodically, and the ß-galactosidase specific activity ((ΔA_420_ per minute per ml of culture per OD_600_) × 1000) was measured and plotted versus the time the sample was harvested relative to the time at which stationary phase for the culture commenced. The strains grown in panel A were BAL4575 (WT; closed squares) and BAL4576 (*ileS(T233P)*; open circles), and strains grown in panel B were BAL4418 (WT; closed squares) and BAL4419 (*ileS(T233P)*; open circles). The data shown are representative of at least three independent experiments.

To determine whether there is a strong decrease in stage-specific sporulation gene expression in the *ileS(T233P)* mutant, we monitored gene expression of Spo0A-controlled genes. Spo0A is the first transcription factor to become active, through phosphorylation by a phosphorelay, in the sporulation regulatory cascade^32^, and if Spo0A-dependent genes have strongly reduced expression, then all subsequent stage-specific sporulation genes will also have reduced expression. One of the operons activated by Spo0A is *spoIIE*, which encodes the phosphatase required for activation of the forespore specific sigma factor, Sigma-F^33^. Thus, we introduced into our isogenic wild-type and *ileS(T233P)* mutant a *spoIIE-lacZ* fusion. Cells were grown in DSM, and samples were collected for ß-galactosidase assay at time points just prior to and for several hours after the entry into stationary phase. As seen in Fig. 3A, while wild-type cells induced *spoIIE* expression one hour after entry into stationary phase, *spoIIE* expression was delayed in the *ileS(T233P)* cells for five hours, after which point *spoIIE* is induced. This late induction of *spoIIE* expression in the *ileS(T233P)* mutant may explain why sporulation does occur in the mutant, albeit at a lower efficiency than wild-type cells.

Altered *spoIIE* expression in the *ileS(T233P)* mutant strain strongly suggests that Spo0A activity is reduced in this mutant strain. To confirm this, we assessed expression of a second Spo0A-activated promoter, *spoIIG*, as a *lacZ* fusion^34^. The *spoIIG* operon encodes the mother cell-specific sigma factor, sigma-E^35^. Similar to what was seen with *spoIIE* expression, expression of *spoIIG* was defective for several hours after entry into sporulation in the *ileS(T233P)* strain (Fig. 3B), confirming the general defect in Spo0A-controlled gene expression.

## Discussion

Here, we present the first strain of *B. subtilis* to lack QC for a mischarged tRNA, specifically tRNA^Ile^. This strain has a point mutation in the endogenous copy of *ileS*, and whole cell extracts of this mutant strain mischarged tRNA^Ile^ with valine unlike the wild-type strain. While this QC-defective strain showed no defect in exponential growth, it has a significantly reduced ability to sporulate. The sporulation defect of the QC-defective IleRS strain appears to be due to weak and/or delayed activation of the first transcription factor in the sporulation cascade, Spo0A. Competition through periods of exponential growth, sporulation, and lethal challenge reveal that the *ileS(T233P)* strain was rapidly outcompeted by the wild-type strain, suggesting that starvation conditions that induce sporulation have selected for the maintenance of the IleRS QC function in *B. subtilis*.

The loss of QC by IleRS in *B. subtilis* leads to a delay in expression of sporulation genes controlled by Spo0A, the first transcription factor that is activated in the sporulation cascade. Spo0A also regulates genes involved in other processes including biofilm formation^26^, but the *ileS(T233P)* strain exhibited a less than two-fold defect in the ability to form biofilms, much smaller than the defect observed for sporulation. Phosphorylation of Spo0A is the rate-limited step for the initiation of sporulation^36^,^37^, and expression of sporulation genes requires a higher level of phosphorylated Spo0A than biofilm genes^38^,^39^, suggesting that the *ileS(T233P)* strain may be defective in forming high levels of phosphorylated Spo0A. Spo0A is activated by phosphorylation through a phosphorelay^40^, and there are several known negative regulators of this phosphorelay. Future research will address what step along this Spo0A activation pathway is affected in the *ileS(T233P)* mutant or whether there is a Spo0A-independent mechanism that is affecting the expression of these *spoII* genes. Understanding how *B. subtilis* controls sporulation has allowed researchers to identify how cells sense cell cycle problems, which are amplified by checkpoints that delay sporulation, including DNA replication, chromosome partitioning, and tricarboycylic cycle metabolism^41^,^42^, and research into how loss of QC by IleRS affects sporulation could reveal a novel checkpoint. It is not known at this time if the requirement of QC for sporulation is specific to IleRS or whether loss of QC by other aaRSs will similarly disrupt sporulation in *B. subtilis*. Intriguingly, the *ileS* gene of *B. subtilis* and other endospore-forming species is adjacent to a gene, *ylyA*, required for spore germination^43^.

What causes sporulation to be so sensitive to the loss of QC by IleRS? IleRS could encounter conditions that promote a higher rate of mischarging of tRNA^Ile^ in stationary phase, through a higher ratio of non-cognate Val to cognate Ile. Alternatively, the rate of mischarging, and subsequent mistranslation, may be identical between exponential growth and stationary phase, and a protein(s) involved in sporulation may be particularly sensitive to mistranslation. A further possibility is that there is a higher ratio of charged to uncharged tRNA^Ile^ as a result of loss of QC by IleRS, and this altered ratio may delay activation of the sporulation pathway. *E. coli* cells expressing an editing-defective form of the phenylalanine tRNA synthetase (PheRS), have a higher ratio of charged to uncharged tRNA^Phe^ under amino acid starvation and are unable to mount a stringent response^44^. In *B. subtilis*, the stringent response is induced by uncharged tRNAs and has been shown to activate sporulation^45^. The higher ratio of charged to uncharged tRNA^Ile^ in *B. subtilis* may also affect the putative ILE-T-Box (i.e. riboswitch) of *ileS*, which could alter the levels of IleRS and possibly exacerbate the problems caused by loss of QC^46^,^47^. Biosynthesis of Ile and other branched-chain amino acids (BCAA) is not predicted to be directly affected in the IleRS QC-defective mutant, as aaRS are highly specific for their cognate tRNA and the BCAA biosynthesis operon *ilvBHC-leuABCD* is controlled by tRNA^Leu^ and a LEU-T-Box^48^. Future experiments to address the potential models outlined here for why quality control by IleRS is critical for sporulation, but not exponential growth, will require measuring cellular amino acid pools, the level of the stringent response alarmone, ppGpp, mistranslation, and charged tRNA^Ile^ in actively growing and sporulating cells.

Sporulation by *B. subtilis* is a developmental process induced by starvation, specifically limitation for carbon, nitrogen, or phosphorous^49^,^50^,^51^, and it is the major state in which *B. subtilis* is isolated from the environment^52^. Cells lacking QC by IleRS are rapidly outcompeted by wild-type cells under conditions requiring sporulation, which strongly argues that sporulation is the physiological condition that selects for the maintenance of QC by IleRS in *B. subtilis.* These data further suggest that the primary role of QC by IleRS in other species, and possibly QC by other aaRSs, may be in allowing survival of cells that have a severely reduced growth rate. Consistent with this, QC by alanine tRNA synthetase (AlaRS) was shown to be required in terminally differentiated mouse neuronal cells and cardiomyocytes, non-growing or slowly-growing cell populations^5^,^53^. Intriguingly, QC by some aaRSs has been lost from mitochondria and *Mycoplasma* species, many of which are intracellular parasites^54^,^55^,^56^,^57^. This latter finding suggests that, in the absence of changing environmental conditions, QC by aaRSs may not be required and is consistent with the suggestion of the work presented here that QC is required by cells that must transition from rapid growth to slow-growth/stationary phase induced by nutrient limitation.

In summary, we have shown for the first time that the quality control function of a tRNA synthetase is critical for sporulation in *B. subtilis.* The IleRS QC was required for activating the genes controlled by the master transcription factor, Spo0A, and cells lacking IleRS QC were rapidly lost from a mixed population when a selection for sporulation was applied. These data suggest that the QC function of tRNA synthetases may be conserved to deal with particular stress conditions that are encountered by cells as their growth slows or they enter stationary phase in response to nutrient limitation.

### Experimental Procedures

#### tRNA Preparation

*Bacillus subtilis* tRNA^Ile^ was transcribed *in vitro* as previously described^58^.

#### Cell Free Extract Preparation

*B. subtilis* tRNA-free cell free extracts were prepared as described^59^, using strains containing either a wild-type *ileS* gene or an editing deficient allele of *ileS (ileS(T233P)*), except EDTA-free protease inhibitor (Roche) was used during lysis to replace PMSF. Briefly, the supernatant following ultracentrifugation was loaded onto DEAE Sephacel (GE) resin. Cell free extracts were quantified using a BCA Protein Assay kit (Thermo Scientific Pierce).

#### Aminoacylation Assays

Aminoacylation assays using cell free extracts of wild-type *ileS* and *ileS(T233P)* strains were performed as previously described^21^. Each reaction contained 450 μg extract protein, 1070 ng/μL of *B. subtilis* tRNA^Ile^, 0.1 mM cognate [^14^C]Ile at 200 cpm/pmol (Moravek Biochemicals), 0.15 mM non-cognate [^14^C]Val at 120 cpm/pmol (PerkinElmer), and 0.3 mM non-cognate [^14^C]Leu at 120 cpm/pmol (Moravek Biochemicals).

#### Growth conditions

*E. coli* and *B. subtilis* strains were routinely grown in Luria-Bertani (LB) medium. For sporulation assays, *B. subtilis* strains were grown in either Difco sporulation medium (DSM)^60^ or S7_50_ minimal medium with 0.1% glucose^61^. Both *E. coli* and *B. subtilis* strains were grown at 37 °C. Antibiotics were used as required at the following concentrations: ampicillin 100 μg/ml, chloramphenicol 5 μg/ml, erythromycin 0.5 μg/ml, and tetracycline 12.5 μg/ml. The growth rate was measured as the slope of the line from the natural log of the OD_600_ readings versus the time of incubation in minutes. Only points in exponential growth were used for a line fit, and only line fits that gave an R-value greater than 0.99 were considered valid.

#### Strain construction

*E. coli* strain Top10 (Invitrogen) was used for the routine construction and maintenance of plasmids. The *B. subtilis* strains used in this study are all derivatives of the JH642 strain, which contains the *trpC2* and *pheA1* mutations^25^ and were constructed by transformation of genetically competent cells with chromosomal DNA or plasmids using standard protocols^60^. Gene replacement was verified using PCR.

The *B. subtilis ileS(T233P)* strain was constructed by transformation with a plasmid, pBL4566, containing the *ileS(T233P)* gene. The pBL4566 plasmid is a derivative of pJet1.2 (Thermo Fisher) that contains a restriction site for the I-SceI meganuclease^62^ and the *cat* gene for chloramphenicol resistance. The *ileS* gene was PCR amplified with primers ileS-F (5′-GCATGGATCCAAGGAATAAATTCTCTGATTA) and ileS-R (5′- GCATAGATCTGCATATCGGTCAACTGAACGC) and ligated to pJet1.2 according to the instructions of the CloneJET PCR Cloning Kit (ThermoFisher Scientific). An I-SceI-*cat* cassette was constructed by PCR amplification with pJH101^63^ as a template and primers I-SceI-*cat-*A (5′-ATGC*CTGCAG***TAGGGATAACAGGGTAAT**TATTGGGCGCTCTTCCGCTAAGCATGCGTTACCCTTATTATCAAGA) and I-SecI-*cat-*B (5′-ATGC*CTGCAG*GCGAGTCAGTGAGCGAGGAAGCAAGCATGCGGAGCTGTAATATAAAAAC), in which the underlined sequence corresponds to the *cat* gene, the I-SecI site is bold, and PstI restriction sites are italicized. Both the I-SceI-*cat* PCR and the pJet1.2-*ileS* plasmid were digested with PstI and ligated, to yield a pJet-*ileS-*I-SceI-*cat* plasmid. This plasmid was subjected to site-directed mutagenesis using primers T233P-F (5′- GCATCATCATTTGGACA**C**CAACGCCGTGGACAATT) and its reverse complement (the bold, underlined nucleotide is the introduced mutation), according to directions of Quikchange Lightning Site-Directed Mutagenesis Kit (Agilent Technologies). The presence of the mutation was confirmed by DNA sequencing, and the resulting plasmid was named pBL4566.

To transfer the *ileS(T233P)* allele on pBL4566 to the chromosome of *B. subtilis*, a wild-type strain of *B. subtilis*, JH642^25^, was transformed with pBL4566, and transformants were selected on plates containing chloramphenicol. The resulting strain, BL4568, which has pBL4566 integrated into its chromosome at the *ileS* locus, has two alleles of *ileS*, one wild-type and one *ileS(T233P)*, flanking the pJet1.2 plasmid backbone and the I-Sce1-*cat* cassette. To return the chromosome to just one copy of *ileS* and remove the pBL4566 plasmid, the pBKJ223 plasmid that expresses the I-Sce1 meganuclease^64^ was transformed into BAL4568, and transformants were selected on plates containing tetracycline. I-Sce1 cleaves the chromosome at the I-SceI restriction site on the pBL4566 plasmid, and repair of this DNA break by homologous recombination results in a strain that has removed the plasmid sequences and restored the native *ileS* chromosomal structure, with the *ileS* allele present being either wild-type or *ileS(T233P)*. To screen for cells that have lost the plasmid sequence, transformants were screened for chloramphenicol sensitivity. The *ileS* allele present in the chloramphenicol-sensitive clones were screened by PCR amplification and DNA sequencing, including the genes to either side of *ileS, ylyA* and *divIVA*. One strain with the *ileS(T233P)* allele and one with the wild-type *ileS* allele were selected. To remove pBKJ223 from the desired clones, cells were grown in LB liquid cultures lacking antibiotic selection. Colonies from these cultures were streak-purified on LB agar plates and then screened for tetracycline sensitivity. Two tetracycline-sensitive clones, one wild-type *ileS* and one *ileS(T233P)*, were named BAL4574 and BAL4571, respectively. To assay expression of sporulation genes, *thrC::(spoIIE-lacZ, erm)*^65^,^66^ and Spß::(*spoIIG-lacZ, cat)*^67^ were introduced into BAL4574 (wild-type) and BAL4571 (*ileS(T233P)*).

#### Sporulation Assays

To assess the *ileS(T233P)* mutant for sporulation, strains were assessed for the presence of heat-resistant spores, as previously described^60^. Briefly, cells were grown in liquid Difco Sporulation Medium (DSM) until 16 hours after the onset of stationary phase. At this time, the total viable CFU/ml was measured and then the cultures were heated at 80 °C for 20 minutes, and the heat-resistant CFU/ml was measured as the number of spores present.

#### β-Galactosidase Assays

Cells were grown as described above for sporulation assays, except that samples were harvested throughout the exponential growth phase and several hours past the entry of the cultures into stationary phase. The cells were then harvested by centrifugation, and resuspended in Z-Buffer, and ß-galactosidase activity was measured as previously described^68^. β-galactosidase-specific activity is presented as (ΔA_420_ per minute per ml of culture per OD_600_) × 1000).

#### Biofilm formation Assays

Cells were grown for 24 hours in polyvinyl chloride microtiter plates in Biofilm Growth Medium, a modified LB based medium supplemented with 124 mM potassium phosphate pH 7.0, 15** **mM ammonium sulphate, 3.4 mM sodium citrate, 0.01 mM MgSO_4_, and 0.1% glucose as previously described^28^,^29^,^30^. After incubation, the wells were washed twice and subsequently stained with crystal violet. The wells were rinsed to remove any non-adherent crystal violet and treated with a solubilizing solution of 80% ethanol, 20% acetic acid; the *A*_570_ of the solution was then measured. The *A*_570_ of 16–24 wells were averaged. When the SEM (standard error of the mean) of the *A*_570_ measurements obtained for the 16–24 wells was <10% of the mean, the assay was considered valid. Each assay was repeated on at least three independent occasions, and the averages from all valid assays were averaged to determine the level of biofilm formation for a strain.

#### Growth of pellicle biofilms

Bacterial cells were inoculated at OD_600_ 0.025 into Biofilm Growth Medium and grown with shaking until OD_600_ ~1.0. The cells were diluted into fresh Biofilm Growth Medium to an OD_600_ of 0.01 and 5 ml were added to culture test tubes. The tubes were incubated at 37 °C for 48 hours with no shaking, at which times the pellicles were imaged.

## Additional Information

**How to cite this article**: Kermgard, E. *et al*. Quality Control by Isoleucyl-tRNA Synthetase of *Bacillus subtilis* Is Required for Efficient Sporulation. *Sci. Rep.* **7**, 41763; doi: 10.1038/srep41763 (2017).

**Publisher's note:** Springer Nature remains neutral with regard to jurisdictional claims in published maps and institutional affiliations.

## Figures and Tables

**Table 1:** Sporulation Frequency of Wild-Type and IleRS(T233P) Cells.

Trial	Wild-Type			IleRS (T233P)		
	Viable^a^	Spores^b^	Frequency^c^	Viable^a^	Spores^b^	Frequency^c^
Difco Sporulation Media						
1	5.9 × 10^8^	3.5 × 10^8^	60%	3.6 × 10^8^	1.3 × 10^8^	35%
2	6.8 × 10^8^	3.8 × 10^8^	56%	3.5 × 10^8^	1.2 × 10^8^	34%
3	4.9 × 10^8^	3.8 × 10^8^	78%	3.2 × 10^8^	1.6 × 10^7^	5%
4	4.6 × 10^8^	3.6 × 10^8^	77%	6.0 × 10^8^	7.1 × 10^7^	12%
5	3.4 × 10^8^	3.5 × 10^8^	100%	3.5 × 10^8^	2.5 × 10^7^	71%
Glucose Minimal Media						
1	2.7 × 10^8^	1.8 × 10^8^	67%	1.6 × 10^8^	3.4 × 10^6^	2.1%
2	2.7 × 10^8^	2.3 × 10^8^	85%	1.8 × 10^8^	1.3 × 10^7^	7.2%
3	9.1 × 10^7^	9.2 × 10^7^	100%	1.1 × 10^8^	3.1 × 10^7^	27%

^a^Reported are the CFU/ml of the cultures after 16 hours of incubation in stationary phase.

^b^After testing for viable cells, the cultures were heat treated at 80 °C for 20 minutes and then plated for CFU/ml counts.

^c^The frequency of sporulation was calculated as [(spores CFU/ml)/(viable CFU/ml)*100].
